# Emergence of a novel porcine pestivirus with potential for cross-species transmission in China, 2023

**DOI:** 10.1186/s13567-025-01472-5

**Published:** 2025-02-07

**Authors:** Li-shuang Deng, Tong Xu, Zhi-wen Xu, Ling Zhu

**Affiliations:** 1 College of Veterinary Medicine, Sichuan Agricultural University, Chengdu, China; 2 Sichuan Key Laboratory of Animal Epidemic Disease and Human Health, Sichuan Agricultural University, Chengdu, China

**Keywords:** *Pestivirus* genus, PAAPeV, swine, phylogenetic analysis, pathogenicity, cross-species transmission

## Abstract

Pestiviruses, RNA viruses belonging to the *Flaviviridae* family, have a broad host range. Their pathogenicity varies greatly and they have caused significant economic losses in animal husbandry. In this study, a novel pestivirus, porcine abortion-associated pestivirus (PAAPeV), was isolated from pigs in China in 2023. Clinically, PAAPeV causes abortions in sows and leads to congenital tremors and death in piglets. PAAPeV replicates efficiently in ST cells. Morphologically, PAAPeV virions are spherical particles with a diameter of approximately 80 nm. Phylogenetic analysis reveals that PAAPeV is closely related to Wenzhou *Pipistrellus abramus* pestivirus and clusters into a distinct branch, suggesting that it represents a new species: *Pestivirus chinensis*. Animal experiments demonstrated that PAAPeV-infected piglets and mice exhibit significant histopathological changes. In piglets, histopathological examination revealed myocarditis, hepatitis, glial vacuolation and cerebrovascular inflammation. In mice, findings included hepatic monocyte aggregation, glomerular atrophy, pulmonary edema, inflammatory cell infiltration, and capillary dilation. Viremia has been detected in both piglets and mice, with high viral genome loads found in various organs. In vitro results revealed that PAAPeV replicates in ST cells and, to a lesser extent, in human (A549 and HepG2) and monkey (Vero) cells. Overall, PAAPeV infects both pigs and mice and has the potential to infect other mammals, indicating its ability for cross-species transmission. This poses significant risks to the pig industry and public health. Strengthening monitoring and prevention efforts for PAAPeV is crucial. Our findings greatly increase the understanding of pestivirus diversity and its broader pathogen spectrum.

**Supplementary Information:**

The online version contains supplementary material available at 10.1186/s13567-025-01472-5.

## Introduction

The *Pestivirus* genus belongs to the *Flaviviridae* family and consists of positive-sense, single-stranded RNA viruses [1]. These viruses have genomes that range in size from 12.3 to 13 kb. Pestiviruses were previously categorized into four species: *Pestivirus bovis* (bovine viral diarrhea virus 1, BVDV1), *Pestivirus tauri* (bovine viral diarrhea virus 2, BVDV2), *Pestivirus suis* (classical swine fever virus, CSFV) and *Pestivirus ovis* (Border disease virus, BDV) [2, 3]. However, owing to the increasing diversity of pestiviruses, the taxonomy of the *Pestivirus* genus was revised in 2017. This revision was based on the analysis of nucleotide and amino acid sequence distances of complete coding sequences (CDSs), alongside considerations of antigenic variations, the natural host range, and associated pathologies. Consequently, pestiviruses were reclassified into 11 species, including seven newly identified species: *Pestivirus antilocaprae* (pronghorn antelope pestivirus, PAPeV), *Pestivirus australiaense* (porcine Bungowannah pestivirus, PPeV), *Pestivirus giraffae* (giraffe pestivirus, GPeV), *Pestivirus brazilense* (HoBi-like bovine pestivirus, HoBiPeV), *Pestivirus aydinense* (Aydin-like pestivirus, AydinPeV), *Pestivirus ratti* (rat pestivirus) and *Pestivirus scrofae* (atypical porcine pestivirus, APPeV) [4]. In a recent study, researchers proposed expanding the number of pestivirus species to 19 on the basis of genetic relationships, as well as information on antigenic properties, host origin, and disease characteristics. This expanded taxonomy introduces eight additional species, including *Pestivirus L* (Linda virus, LindaV), *Pestivirus M* (Phocoena pestivirus, PhoPeV), *Pestivirus N* (Tunisian sheep-like pestivirus, TSV), *Pestivirus O* (ovine/IT pestivirus, ovIT-PeV), *Pestivirus P* (Dongyang pangolin virus, DYPV), *Pestivirus Q* (rodent pestivirus, RtNn-PeV), *Pestivirus R* (rodent pestivirus, RtAp-PeV) and *Pestivirus S* (bat pestivirus, BtSk-PeV) [5].

The pestivirus genome contains a single open reading frame (ORF) that encodes a polyprotein flanked by 5′ and 3′ untranslated regions (UTRs). This polyprotein is cleaved by both cellular and viral proteases, producing four structural proteins (C, E^rns^, E1, E2) and eight non-structural proteins (N^pro^, P7, NS2, NS3, NS4A, NS4B, NS5A, NS5B) [6]. Among these, the 5′ UTR is highly conserved across pestiviruses and often serves as a crucial element for strain classification [7, 8]. In addition, the N-terminal protease (N^pro^) and envelope glycoproteins E2 and NS3 are commonly used in phylogenetic analyses [9–11]. Notably, E^rns^, E2 and NS3 elicit measurable humoral immune responses in hosts following natural infection [12]. In classical pestiviruses such as CSFV and BVDV, E2 functions as an immune-dominant antigen, acting as the primary target for neutralizing antibodies [13–15]. E^rns^, an envelope glycoprotein with RNase activity, and N^pro^, known for its autoproteolytic activity, exhibit unique characteristics specific to the *Pestivirus* genus [16].

Pestiviruses have a remarkable ability to infect various hosts, including swine, ruminants, bats and rodents, causing significant economic impacts on domestic animals [5, 17, 18]. Diseases associated with pestivirus infections include a range of conditions, such as hemorrhagic symptoms, abortion, respiratory disorders, gastroenteritis and immune system dysfunction. Notably, these viruses exhibit two distinct biotypes, cytopathogenic (cp) and non-cytopathogenic (noncp), distinguished by their ability or inability to cause cytopathic effects (CPEs) in cell culture. The majority of pestiviruses are noncp variants [4].

In this study, we successfully isolated a novel pestivirus from aborted fetuses and piglets with congenital tremors at a pig farm in China. We investigated the genetic characteristics and cellular tropism of this newly identified virus. Additionally, we conducted animal infection experiments to explore its pathogenicity and tissue distribution. We aim to increase awareness of this emerging pestivirus and enhance the understanding of the pathogen spectrum and host range of pestiviruses. This will, in turn, contribute to advancements in veterinary and public health knowledge.

## Materials and methods

### Clinical samples

From April to May 2023, a pig farm in Sichuan Province, China, reported an unexplained epidemic. The outbreak initially presented as reproductive disorders in sows, including abortions and the birth of stillborn and mummified fetuses. This reproductive failure was followed by the emergence of congenital tremors and death in the piglets. The prevalence of congenital tremors in piglets is approximately 70%, with an overall mortality rate of approximately 20%. The farm veterinarians treated the pigs with various broad-spectrum antibiotics, but none were effective. Clinical samples were collected from aborted fetuses and piglets exhibiting congenital tremors. Comprehensive PCR and RT-PCR were conducted to detect common viral pathogens, including APPeV, CSFV, porcine reproductive and respiratory syndrome virus (PRRSV), porcine pseudorabies virus (PRV), porcine circovirus type 2 (PCV2) and porcine parvovirus (PPV). All the samples were stored at -80 ℃ for further analysis.

### Viral metagenomic analysis

The collected samples were sent to Chengdu Life Baseline Technology Co., Ltd. (Chengdu, China) for virus metagenomic sequencing to investigate the causes of this outbreak. Specifically, a 10% tissue suspension in phosphate-buffered saline (PBS) was centrifuged, filtered, and treated with DNase and RNase to digest unprotected nucleic acids. Total nucleic acids were then extracted using a QIAamp Viral RNA Mini Kit (Qiagen, Hilden, Germany) and reverse transcribed into cDNA using SuperScript III Reverse Transcriptase (Invitrogen, Carlsbad, CA, USA). Thirty-two libraries were prepared and sequenced on an Illumina NovaSeq platform (Illumina, San Diego, CA, USA). The data were debarcoded, processed, cleaned, and assembled using VecScreen in the National Center for Biotechnology Information (NCBI) [19] and Geneious Prime v2019.2.3 (Biomatters, Auckland, New Zealand). Contigs and singlets were matched against an in-house viral proteome database using BLASTx with an E value of < 10^–5^.

### Viral isolation

Swine testis (ST) cells were cultured in 6-well plates with Eagle’s minimum essential medium (MEM; Gibco, Grand Island, NY, USA) supplemented with 10% fetal bovine serum (FBS; Gibco, Grand Island, NY, USA) until 80% confluency. Positive sample supernatants were then inoculated onto the cells and incubated at 37 °C with 5% CO_2_ for 1 h. Then, the supernatants were replaced with MEM supplemented with 2% FBS, 100 U/mL penicillin and 0.1 mg/mL streptomycin. The isolated virus was subsequently passaged in ST cells repeatedly until stable viral propagation was attained.

### Establishment of a TaqMan probe-based RT-qPCR assay

A TaqMan probe-based RT-qPCR assay was established for the detection of this virus. Briefly, specific primers (NS3-WTF, 5ʹ-CACGGAGCTACCAAGGAAAT-3ʹ; NS3-WTR, 5ʹ-GGAAATGCTAGGGTGCTTAAC-3ʹ) targeting the NS3 gene of the virus were designed to amplify a 127-bp fragment using Primer Premier 5.0 software (PREMIER Biosoft International, Palo Alto, CA, USA). The TaqMan probe 5ʹ-TGGAAGCAATAGGGAGGCACAAGA-3ʹ was labelled with the fluorescent reporter dye VIC at the 5ʹ end and with the nonfluorescent quencher BHQ1, associated with a minor groove binder, at the 3ʹ end. Viral RNA was extracted from the virus culture supernatant using the RNAiso plus reagent (Takara, Dalian, China). The RNA was subsequently reverse transcribed to cDNA using the PrimeScriptTM RT Reagent Kit (Takara, Dalian, China). A 127-bp fragment of the virus NS3 gene was amplified and cloned and inserted into the pMD-19T vector (Takara, Dalian, China) to construct the plasmid pMD-NS3. The RT-qPCR reaction mixture (25 μL) included 12.5 μL of Premix Ex Taq (Probe RT-qPCR) (Takara, Dalian, China), 1 μL (10 μM) of each primer, 0.2 μL (10 μM) of probe, 2 μL of cDNA, and 8.3 μL of H_2_O. The experiment was performed on a QuantStudio 1 Plus qPCR system (Thermo Fisher, ABI, China) with the following conditions: 50 °C for 2 min, 95 °C for 5 min, followed by 40 cycles of 95 °C for 20 s and 60 °C for 1 min.

### Preparation of mouse antiserum against the E2 protein

To produce antiserum against this virus, the full-length E2 protein was expressed using a prokaryotic expression system. Initially, the full-length CDS encoding the E2 protein was amplified using the primer pair E2-F (5ʹ-CCG*CTCGAG*CGGGGCCTAGAATGTAATCATG-3ʹ) and E2-R (5ʹ-TGC*TCTAGA*GCATTGGTCAGCCATGACTTG-3ʹ). The full-length E2 gene was subsequently cloned and inserted into the pCold-TF vector using the *Xho*I and *Xba*I enzymes (Takara, Dalian, China) to construct a recombinant expression plasmid (pCold-TF-E2). The recombinant plasmid (pCold-TF-E2) was then transformed into *E. coli* BL21 (DE3; Sangon Biotech, Shanghai, China) for protein production. After induction with IPTG, the recombinant His-E2 protein was purified using Ni–NTA affinity chromatography (Sangon Biotech, Shanghai, China). The purified protein was identified by SDS-PAGE and western blot analysis. Six-week-old female BALB/c mice were immunized with purified protein (100 μg/dose) mixed with Freund’s complete adjuvant. Booster immunizations were then administered, and antiserum was collected 1 week after the third booster.

### Indirect immunofluorescence assay

Infected ST cells were fixed with 4% paraformaldehyde for 15 min and permeabilized with 0.1% Triton X-100 for 15 min. The cells were then incubated with mouse antiserum against E2 protein (1:200) prepared in this study for 2 h at room temperature, followed by incubation with CoraLite488-conjugated goat anti-mouse IgG (1:1000; Proteintech, Chicago, IL, USA) as the secondary antibody for 1 h. Nuclei were stained with DAPI (Beyotime, Shanghai, China) for 5 min. After each step, the cells were washed three times with PBS. Observations were made using a Leica fluorescence microscope (Leica, Wetzlar, Germany).

### Electron microscopy

After 24 h of viral inoculation, infected ST cells were fixed in 2.5% glutaraldehyde and then prepared into ultrathin sections for observation using a JEM-1400FLASH electron microscope (JEOL, Tokyo, Japan).

### Phylogenetic analysis of the virus

To obtain the viral genome sequence, we designed ten primer pairs (Table 1) on the basis of the contigs from the metagenomic sequencing. These primers were used to amplify overlapping fragments via PCR. The 5′ and 3′ termini of the viral genome were determined using the SMARTer RACE 5'/3' Kit (Clontech, Beijing, China) following the manufacturer’s instructions. The purified PCR products were subsequently cloned and inserted into pMD-19T vectors, and three positive clones from each fragment were sent to Sangon Biotech Co., Ltd. (Shanghai, China) for Sanger sequencing. SeqMan software (DNASTAR Inc., WI, USA) was used for sequence assembly, and MEGA software (Mega Limited, Auckland, New Zealand) and MegAlign software (DNASTAR Inc., WI, USA) were employed for sequence alignment. Phylogenetic trees were constructed using the maximum-likelihood method and the Kimura two-parameter model in MEGA software. Bootstrap analyses were performed with 1000 replicates.

**Table 1:** **Primers used in this study for whole-genome amplification of PAAPeV**

Prime	Sequence (5ʹ-3ʹ)	Location in genome (nt)	Source or reference
1F	TCTTTGTAGGAAAATTGGGG	1–20	This study
1R	GTCAGGATTGTTGGTGTC	1581–1598	This study
2F	GCATGGATGGTGTAATTGG	1425–1443	This study
2R	ATGCCACTGTATCTGCTTG	3046–3064	This study
3F	CAGTGTGTCATAACCAGCAG	2866–2885	This study
3R	TAGTGTGTGTACGCCTTCTG	4415–4434	This study
4F	GCTTACTTAACCGCATTGAC	4156–4175	This study
4R	TGCGTGCCTGACATGATTG	5735–5753	This study
5F	GTTAAGACGGACTCTGGTTG	5470–5489	This study
5R	TTCTGCATGTCATCAGCTCG	7050–7069	This study
6F	CTACCACTACGACTTGCTAC	6744–6763	This study
6R	TTCATCAGTGGCAGATTGG	8199–8217	This study
7F	AATTACCTGCCTTACGCTG	7903–7921	This study
7R	TATGACGGTGACATTCTTGG	9374–9395	This study
8F	TGAGAGAAGGGCTTGTACC	9116–9134	This study
8R	CAGCTCACCTCCTCATAG	10,560–10,577	This study
9F	CCTATAGTGTCAGAGAGGAAG	10,318–10,338	This study
9R	TGTGTGCCTGCTATATATTCC	11,815–11,835	This study
10F	GAATCTGTCTAATAGTGATG	11,633–11,652	This study
10R	ACAGAATAGAATAGAATAGAATAG	12,551–12,574	This study
5' RACE	TCCTCTCACTCGCATCCAAC	1362–1381	This study
3' RACE	GGAGGTGAGCTGGGATGTACTT	11,147–11,168	This study

### Experimental infection of human and monkey cell lines

Human cell lines (A549 and HepG2) and a monkey embryonic kidney cell line (Vero) were infected with the virus at a concentration of 9.5 × 10^10^ viral genome copies/mL. After incubation for 1 h, the cells were cultured in Dulbecco’s modified Eagle medium/nutrient mixture F-12 (DMEM/F12) or Dulbecco’s modified Eagle’s medium (DMEM) supplemented with 2% FBS for 48 h at 37 °C in a 5% CO_2_ incubator. The infected cells were subjected to three cycles of freezing and thawing, and the collected supernatants were centrifuged at 12 000 rpm for 10 min to obtain viral stocks. These stocks were then used to infect the same cell lines, and the viral RNA genome load of each passage was determined by RT-qPCR.

### Experimental infection of animals

To investigate the pathogenicity of the virus isolated in this study, BALB/c mice and piglets were experimentally infected. The mice were purchased from Beijing Huafukang Biotechnology Co., Ltd. (Beijing, China), and the piglets were purchased from Sichuan Meishan Wanjiahao Pig Breeding Co., Ltd. (Meishan, China). Six-week-old BALB/c mice were injected intramuscularly with 200 μL of virus (3.70 × 10^12^ viral genome copies/mL), whereas the control group received 200 μL of MEM. One-week-old healthy piglets that were confirmed to be negative for PRV, PCV2, PPV, APPeV, CSFV and PRRSV were randomly assigned to the infection and control groups. The infected piglets received 2 mL of virus (3.70 × 10^12^ viral genome copies/mL) administered intramuscularly, whereas the control piglets received 2 mL of MEM. Serum samples were collected from piglets and mice at 1–7 days post-infection (dpi). The temperature data and clinical signs were recorded daily. Dead animals were immediately dissected, and surviving animals were humanely euthanized for further investigation. Tissue samples from various organs were collected for analysis of viral tissue distribution. Briefly, 0.1 g of tissue was homogenized in PBS to prepare a 10% tissue suspension. The homogenates were then clarified by centrifugation at 12 000 rpm for 3 min, and the supernatants were used for RNA extraction. This processed RNA served as the template for RT-qPCR analysis.

### Histopathological and immunohistochemistry analysis

Tissue samples from infected piglets and mice were collected. Hematoxylin and eosin (H&E) staining and immunohistochemistry (IHC) analysis were performed following established protocols [20]. Mouse antiserum against the E2 protein was used as the primary antibody for IHC.

### Statistical analysis

The data are presented as the mean ± standard deviation (SD) and were visualized using GraphPad Prism software (GraphPad Software, Inc., USA). Statistical analysis was performed using a two-way analysis of variance (ANOVA), followed by multiple comparisons in GraphPad Prism. A *P* value < 0.05 was considered statistically significant, with significance levels indicated as follows: *P < 0.05; ns, not significant.

## Results

### Sample collection

From April to May 2023, a pig farm in Sichuan Province, China, reported an unexplained epidemic characterized by sow abortions and the birth of stillborn and mummified fetuses (Additional files 1A and B). Affected piglets exhibited congenital tremors and died (Additional file 1C). Autopsies of these piglets revealed significant damage to the lungs, spleen, and liver (Additional files 1D–F). Despite extensive testing, including PCR and RT-PCR assays, no known viral pathogens have been identified.

### High-throughput sequencing and assembly

In this study, virus metagenomic sequencing of lung and brain tissue samples from affected piglets revealed a novel pestivirus (Additional file 2) closely related to the Wenzhou *Pipistrellus abramus* pestivirus 1 isolate YJB_Pabr (GenBank accession No. OM030320.1). The complete viral genome sequence was verified by RT-PCR and Sanger sequencing of cDNA segments, with the primer sequences listed in Table 1. This newly identified virus, designated porcine abortion-associated pestivirus (PAAPeV), has its complete genome sequence deposited in GenBank under accession No. PP663643.1.

### Isolation and identification of PAAPeV

**Figure 1:**
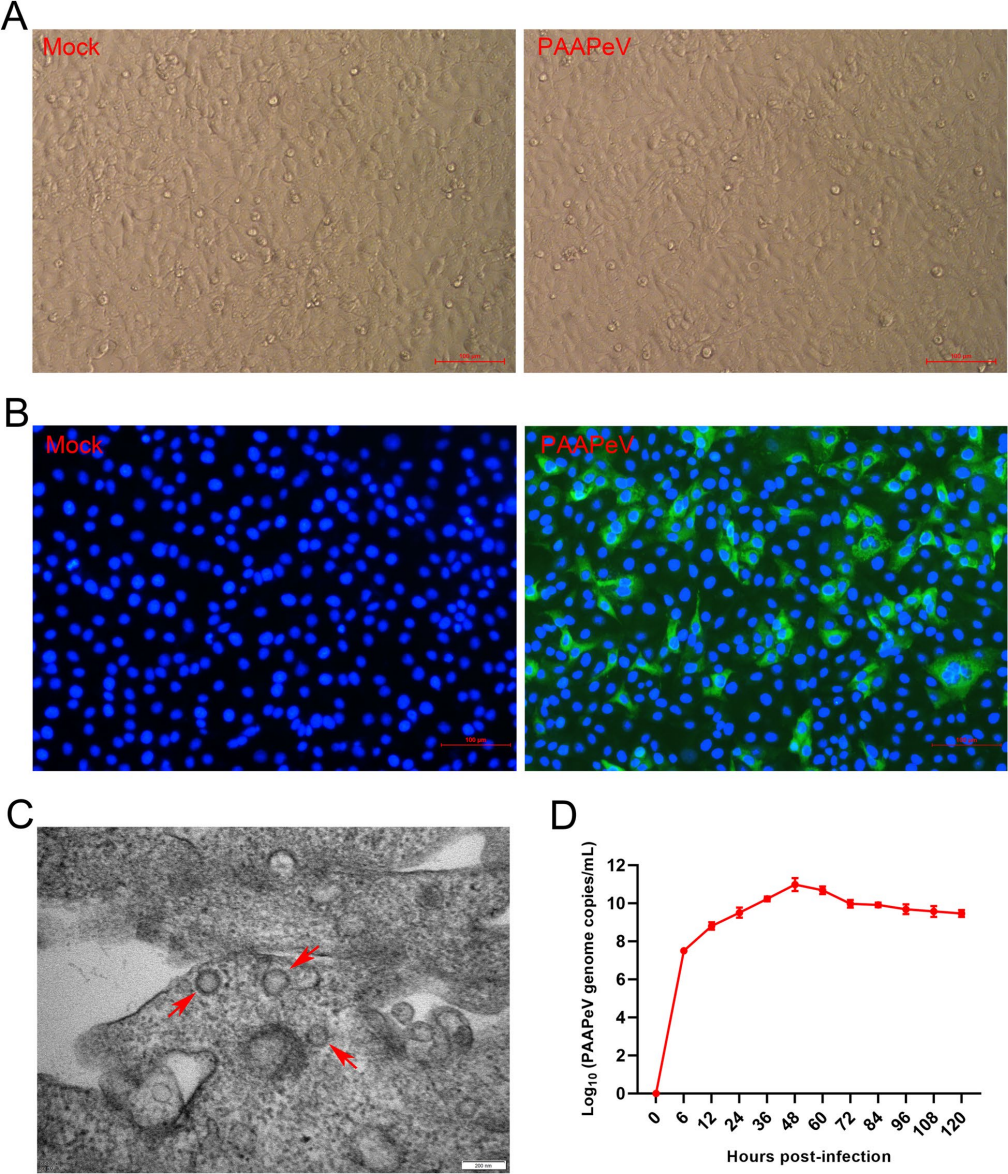
**Growth characteristics of PAAPeV in ST cells**. **A** Image showing PAAPeV infection in ST cells, with no significant CPEs observed at 48 h post-infection (hpi). The control cells were treated with MEM. Magnification: 100×. **B** Detection of the PAAPeV E2 protein in ST cells at 48 hpi via an indirect immunofluorescence assay (IFA). Magnification: 100×. **C** Electron microscopy image of PAAPeV particles in ultrathin cell sections (indicated by red arrows). Magnification: 80 000×. **D** Growth dynamics of PAAPeV in the 15^th^ passage. Viral genome copy numbers were measured by RT-qPCR.

PAAPeV was serially passaged in ST cells for more than 20 generations, resulting in strong detection of viral nucleic acids with no observable CPEs (Figure 1A). A standard curve for the TaqMan RT-qPCR assay was established (y = − 3.4686x + 46.009, R^2^ = 0.9998) (Additional file 3). RT-qPCR was then utilized for PAAPeV detection. Further validation was carried out using indirect immunofluorescence assay (IFA) and TEM. The IFA results revealed positive green fluorescence in the infected cells (Figure 1B). The primary antibody used was mouse antiserum against the E2 protein (1:200), which was prepared in this study (Additional file 4). The TEM results revealed that the PAAPeV virions were spherical particles with an approximate diameter of 80 nm (Figure 1C). The one-step growth curve of PAAPeV in ST cells demonstrated a peak viral genome load (1.18 × 10^11^ PAAPeV genome copies/mL) at 48 h (Figure 1D).

### Genome sequence and phylogenetic analysis of PAAPeV

**Figure 2:**
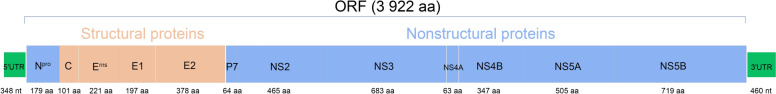
**Schematic representation of the PAAPeV genome**. Nucleotide (nt) or amino acid (aa) lengths are indicated by numbers. The orange squares denote structural proteins, the blue squares represent non-structural proteins, and the green squares indicate untranslated regions (UTRs).

The PAAPeV genome spans 12 574 nucleotides (nt), consisting of a 5ʹ UTR (348 nt, positions 1 to 348), a single ORF (11 766 nt, positions 349 to 12 114), and a 3ʹ UTR (460 nt, positions 12 115 to 12 574), as predicted by the ORF finder tool in the NCBI [19]. On the basis of known pestiviruses, we annotated the presumed proteins encoded by PAAPeV, including N^pro^ (179 amino acids (aa), positions 1–179), C (101 aa, positions 180–280), E^rns^ (221 aa, positions 281–501), E1 (197 aa, positions 502–698), E2 (378 aa, positions 699–1076), P7 (64 aa, positions 1077–1140), NS2 (465 aa, positions 1141–1605), NS3 (683 aa, positions 1606–2288), NS4A (63 aa, positions 2289–2351), NS4B (347 aa, positions 2352–2698), NS5A (505 aa, positions 2699–3203) and NS5B (719 aa, positions 3204–3922). A structural map of the PAAPeV genome is shown in Figure 2.

**Figure 3:**
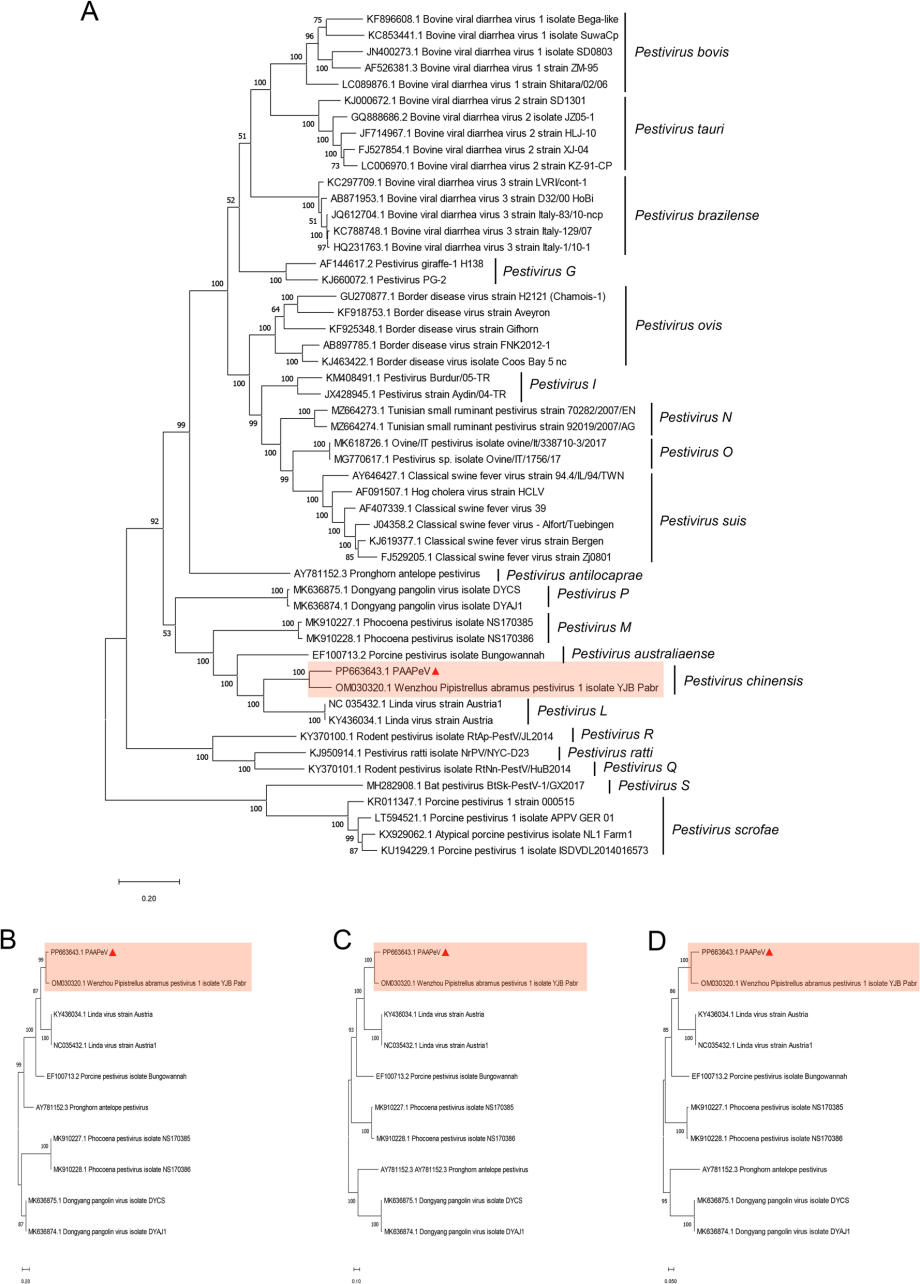
**Phylogenetic analysis of PAAPeV compared with representative pestiviruses downloaded from NCBI. A** Phylogenetic tree of PAAPeV alongside 51 representative pestiviruses constructed from the sequences of the complete open reading frame (ORF). **B**–**D** Phylogenetic trees of PAAPeV and 9 representative pestiviruses based on the sequences of the N^pro^, E2 and NS3 genes. All phylogenetic trees were constructed using MEGA software with 1000 bootstrap replicates.

Phylogenetic analysis of the ORF nucleotide sequences of PAAPeV, alongside 51 other pestivirus sequences downloaded from NCBI (Additional file 5), revealed that PAAPeV has the highest identity with Wenzhou *Pipistrellus abramus* pestivirus, which clusters into a distinct branch. Moreover, PAAPeV is closely related to Linda viruses (GenBank accession Nos. NC_035432.1 and KY 436034.1) and Bungowannah virus (GenBank accession No. EF100713.2) (Figure 3A). The phylogenetic analysis of the N^pro^, E2 and NS3 genes also consistently revealed that PAAPeV has the closest relationship with Wenzhou *Pipistrellus abramus* pestivirus (Figures 3B–D). Additionally, ORF nucleotide identity analysis revealed that PAAPeV shares 86.9% identity with Wenzhou *Pipistrellus abramus* pestivirus, 70.6% identity with Linda virus and 66.7% identity with Bungowannah virus (Additional file 6A). The ORF nucleotide identity between PAAPeV and other pestiviruses does not exceed 65%. Similarly, PAAPeV shares 93.6% amino acid identity with Wenzhou *Pipistrellus abramus* pestivirus, 76.8% amino acid identity with Linda virus and 70.8% amino acid identity with Bungowannah virus (Additional file 6B). The ORF amino acid identity between PAAPeV and other pestiviruses is also less than 65%. Nucleotide and amino acid identities for individual genes between PAAPeV and Linda virus or Bungowannah virus are shown in Table 2. These results indicate that the nucleotide and amino acid identities of genes between PAAPeV and Wenzhou *Pipistrellus abramus* pestivirus range from 84.3 to 93.1% and 87.6 to 98.4%, respectively. The nucleotide identities of genes between PAAPeV and Linda virus ranged from 62.1 to 75.7%, while the amino acid identities ranged from 62.1 to 90.5%. Similarly, the nucleotide identities of genes between PAAPeV and Bungowannah virus ranged from 54.7 to 80.4%, with amino acid identities ranging from 55.4 to 87.3%. The 5' UTR of PAAPeV shares 76.3% identity with Linda virus and 68.7% identity with Bungowannah virus, whereas the 3ʹ UTR shares 72.5% identity with both Linda virus and Bungowannah virus. The results of the multisequence alignment of the UTRs are presented in Additional file 7.

**Table 2:** **Nucleotide and amino acid identities of PAAPeV strains compared with the four most closely related pestivirus strains**

Region	Shared nt identity (%)/aa identity (%)			
	Wenzhou *Pipistrellus abramus* pestivirus	Linda virus 1	Linda virus 2	Bungowannah virus
5' UTR	7.2/–	76.3/–	76.3/–	68.7/–
N^pro^	85.5/88.5	63.7/64.8	63.7/	60/58.7
C	85.5/91.1	74.9/81.2	74.9/81.2	74.1/82.8
E^rns^	85.2/90	71.8/80.5	71.8/80.5	70.1/77.4
E1	87/92.9	67.7/73.6	67.7/73.6	64.5/65.8
E2	84.3/87.6	62.1/62.1	62.1/62.1	57.2/56.1
P7	90.6/95.3	62.5/62.5	62.5/62.5	54.7/59.4
NS2	85.4/94	67/69.7	67/69.7	63.7/64.9
NS3	87.4/96.8	74.7/87.4	74.7/87.4	73.6/85.1
NS4A	93.1/98.4	75.7/90.5	75.7/90.5	80.4/87.3
NS4B	87.9/97.4	75.2/85.3	75.2/85.3	68/75.5
NS5A	86.1/91.9	68.1/70.4	68.1/70.4	59.4/55.4
NS5B	87.3/94.6	70.8/78.5	70.8/78.5	68.1/73.1
3' UTR	66.9/–	72.5/–	72.5/–	72.5/–
ORF	86.9/93.6	70.6/76.8	70.6/76.8	66.7/70.8

nt: nucleotide, aa: amino acid, UTR: untranslated region, ORF: open reading frame, – not translated into protein

### Replication of PAAPeV in human and monkey cell lines

**Figure 4:**
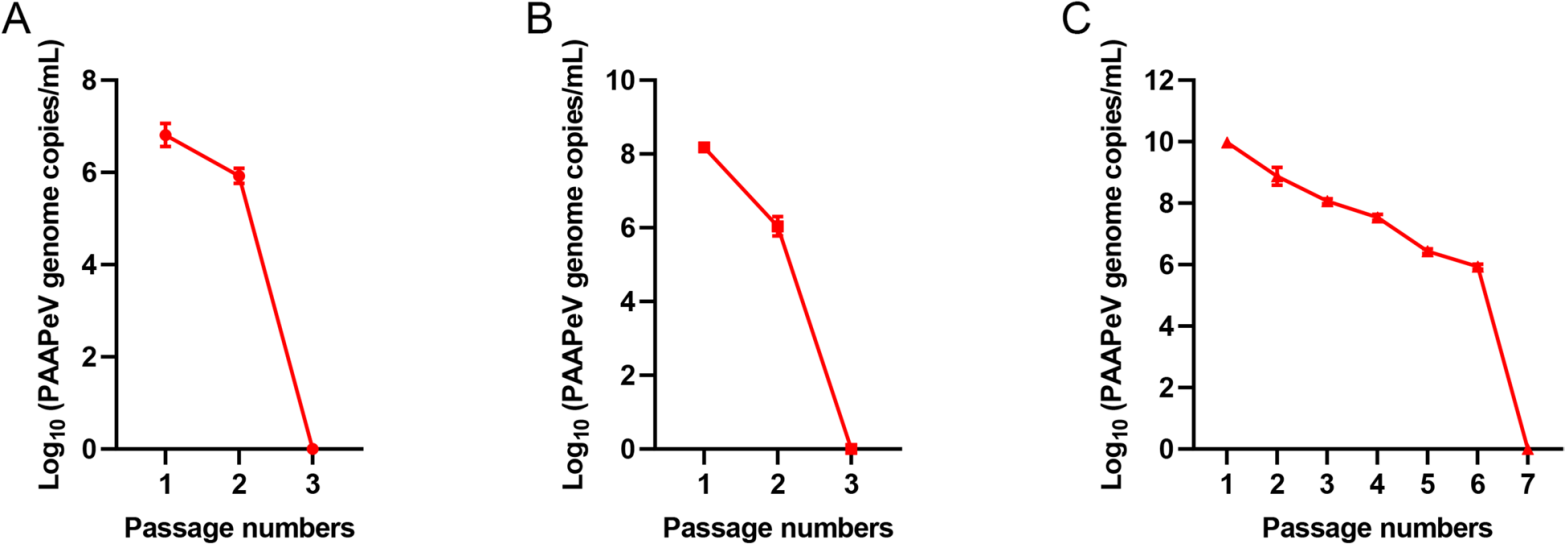
**Replication of PAAPeV in human and monkey cell lines.** Viral genome copy numbers were measured by RT-qPCR assay and are presented as log_10_ values. **A** PAAPeV replication in A549 cells. **B** PAAPeV replication in HepG2 cells. **C** PAAPeV replication in Vero cells.

To investigate PAAPeV tropism in human and monkey cell lines, its replication was assessed in A549, HepG2 and Vero cells. Notably, PAAPeV exhibited limited replication in A549 and HepG2 cells, sustaining only two generations, with viral nucleic acid becoming undetectable by the third generation (Figures 4A and B). Conversely, PAAPeV exhibited continuous replication in Vero cells for up to six generations, with viral nucleic acid becoming undetectable by the seventh generation (Figure 4C). These findings highlight the differential permissiveness of PAAPeV across human and monkey cell lines, with Vero cells supporting more sustained viral replication.

### Pathological lesions in piglets experimentally infected with PAAPeV

**Figure 5:**
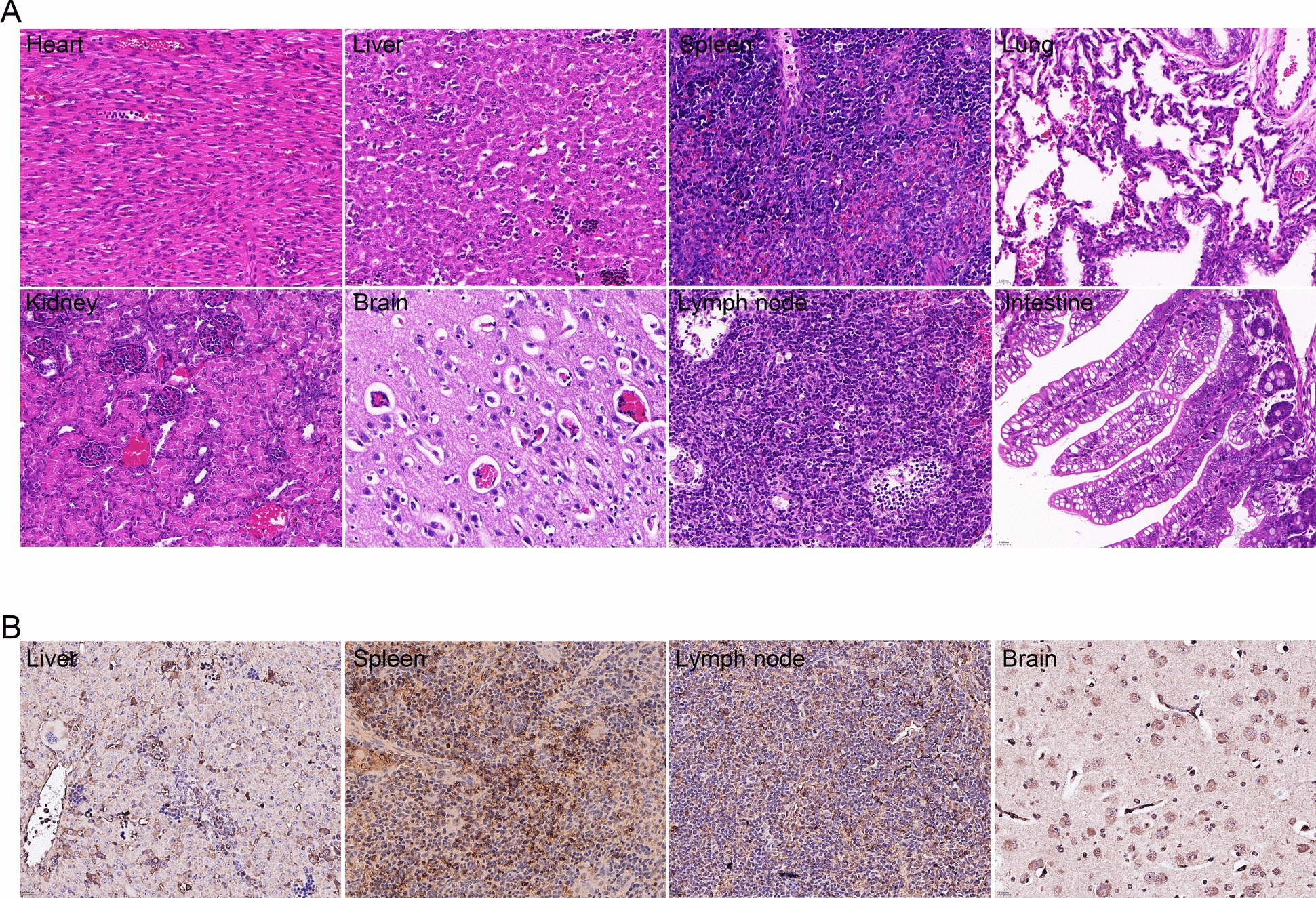
**Histological changes in the organs of piglets infected with PAAPeV**. **A** H&E staining of the heart, liver, spleen, lungs, kidney, brain, lymph nodes and intestine of piglets infected with PAAPeV. Magnification: 400×. **B** Immunohistochemistry (IHC) analysis of the liver, spleen, lymph nodes and brain of piglets infected with PAAPeV. Magnification: 400×.

The rectal temperature of infected piglets significantly increased at 2 dpi, but no noticeable clinical symptoms were observed. At 7 dpi, three infected piglets were euthanized, revealing pathological changes in multiple organs, including the lungs, liver, spleen, kidney and lymph nodes. These changes were characterized by pulmonary interstitial lesions, liver discolouration, splenomegaly, and lymph node haemorrhages and pinpoint haemorrhages in the kidney (Additional file 8). Histopathological examination of various organs revealed multifocal, nonsuppurative myocarditis and hepatitis; lymphocytopenia; extramedullary haematopoiesis in the spleen; capillary congestion in the alveolar walls; vacuolation of glial cells in the brain; and decreased parenchymal cell counts in the lymph nodes accompanied by haemorrhage (Figure 5A). PAAPeV infection was also visualized in the liver, spleen, lymph nodes and brain of infected piglets via an IHC assay with mouse antiserum against the E2 protein (1:200) (Figure 5B).

### Viremia and tissue tropism in piglets infected with PAAPeV

**Figure 6:**
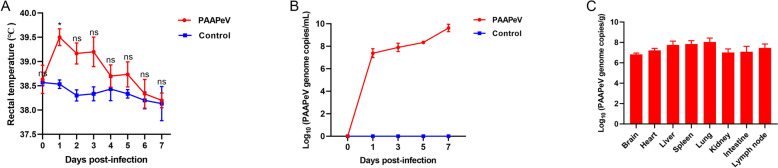
**Changes in rectal temperature and virus detection in the blood and organs of piglets infected with PAAPeV. A** Changes in rectal temperature in piglets infected with PAAPeV. *P < 0.05; ns, not significant. **B** Detection of viremia in piglets infected with PAAPeV. **C** Distribution of PAAPeV in various organs of infected piglets.

Piglet rectal temperatures were monitored daily and increased from 2 to 4 dpi, with the peak occurring at 2 dpi (Figure 6A). Serum samples collected at 1, 3, 5 and 7 dpi presented increasing viral genome loads throughout the infection period, with a notable significant increase at 7 dpi, indicating PAAPeV-induced viremia in infected piglets (Figure 6B). In addition, PAAPeV was detected in various organs, including the brain, heart, liver, spleen, lungs, kidney, intestine and lymph nodes, with the highest viral genome load observed in the lungs (1.45 × 10^8^ PAAPeV genome copies/g), followed by the spleen (8.24 × 10^7^ PAAPeV genome copies/g) and the liver (7.30 × 10^7^ PAAPeV genome copies/g) (Figure 6C).

### Experimental PAAPeV infection in mice

**Figure 7:**
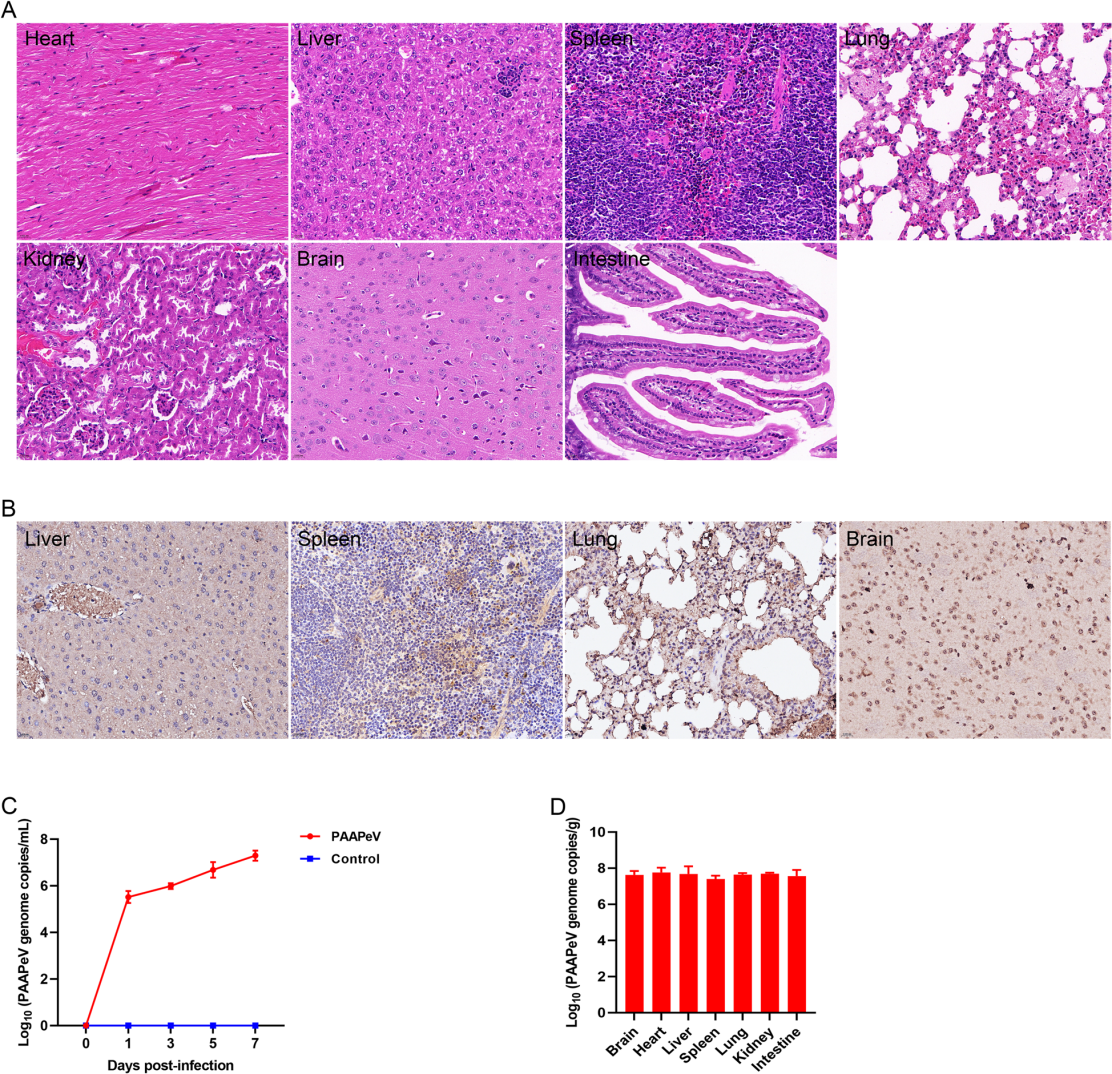
**Histological changes and virus detection in the blood and organs of mice infected with PAAPeV. A** H&E staining of the heart, liver, spleen, lungs, kidney, brain and intestine of mice infected with PAAPeV. Magnification: 400×. **B** IHC analysis of the liver, spleen, lungs and brain of mice infected with PAAPeV. Magnification: 400×. **C** Detection of viremia in mice infected with PAAPeV. **D** Distribution of PAAPeV in various organs of infected mice.

BALB/c mice infected with PAAPeV presented no clinical signs from 1 to 7 dpi, and euthanasia was performed at 7 dpi. Although no visible lesions were observed upon dissection (Additional file 9), histopathological analysis revealed monocyte aggregation in the liver and glomerular atrophy, alongside pulmonary edema, inflammatory cell infiltration, capillary dilatation, and fibroid hyperplasia in the alveolar cavity (Figure 7A). IHC analysis confirmed the presence of PAAPeV in the liver, spleen, lungs and brain (Figure 7B). The viral genome loads in mouse blood at 1, 3, 5 and 7 dpi gradually increased as the infection progressed (Figure 7C). Additionally, viral tissue distribution analysis revealed the presence of PAAPeV in all the examined organs, with higher viral genome loads detected in the heart (6.52 × 10^7^ PAAPeV genome copies/g) and the liver (6.16 × 10^7^ PAAPeV genome copies/g) than in the other organs (Figure 7D). Taken together, these findings demonstrate the replication capability of PAAPeV in mice.

## Discussion

The *Pestivirus* genus belongs to the *Flaviviridae* family and comprises positive-sense, single-stranded RNA viruses. Pestiviruses have a broad host range and cause significant economic losses in domestic animals. From April to May 2023, an unknown disease outbreak occurred at a pig farm in Sichuan, leading to abortions, stillbirths, and mummified fetuses in sows, as well as congenital tremors and deaths in piglets. During the outbreak, the farm veterinarian administered various broad-spectrum antibiotics, but these treatments were ineffective. To confirm the presence of a viral pathogen, we collected clinical samples for metagenomic sequencing. The sequencing identified a virus potentially related to sow abortions and congenital tremors in piglets, which we named PAAPeV. On the basis of the viral sequences, we established a TaqMan probe-based RT-qPCR assay for large-scale and frequent testing on farms. Positive piglets were culled, and positive sows were isolated. Ultimately, the outbreak was brought under control, and we found that PAAPeV persisted in sows for 90 to 110 days. In addition, we successfully isolated the virus from clinical samples, and PAAPeV demonstrated robust replication in ST cells.

Phylogenetic analysis of the ORF sequence of PAAPeV, as well as its N^pro^, E2 and NS3 genes, revealed a close genetic relationship with the Wenzhou *Pipistrellus abramus* pestivirus, which clustered them into a single branch. Further comparison of ORF nucleotide and amino acid identities revealed that PAAPeV has the highest identity with the Wenzhou *Pipistrellus abramus* pestivirus, with a nucleotide identity of 86.9% and an amino acid identity of 93.6%. PAAPeV shares a nucleotide identity of 70.6% with the closely related Linda virus and 66.7% with the Bungowannah virus, along with amino acid identities of 76.8% and 70.8%, respectively. For other pestiviruses, both nucleotide and amino acid identities are less than 65%. The results for individual genes are consistent with those observed for the ORF. According to the taxonomy ratified by the International Committee on Taxonomy of Viruses (ICTV) [21], PAAPeV qualifies as a distinct pestivirus species. Building on previous proposals that classified pestiviruses into 19 species [5], we suggest that PAAPeV and Wenzhou *Pipistrellus abramus* pestivirus be designated as new species within the *Pestivirus* genus: *Pestivirus chinensis*.

PAAPeV causes notable pathological changes in the organs of piglets, particularly in the liver, spleen and lungs. Pathological analysis revealed multifocal, nonsuppurative myocarditis and hepatitis, along with cerebral glial cell vacuolation and inflammation in cerebral vessels. These lesions closely resemble those associated with Linda virus and Bungowannah virus infections [22–25]. IHC analysis further confirmed the presence of PAAPeV in brain neurons, similar to the presence of Linda virus in trigeminal nerve neurons [23]. In addition, high PAAPeV genome loads were detected in all organs, accompanied by viremia. Mice infected with PAAPeV displayed no obvious clinical signs. However, pathological damage was observed in the liver, kidney, and lungs. Additionally, PAAPeV was detected in the blood and organs. These results suggest that PAAPeV can successfully infect mice and has the potential for cross-species transmission.

Although PAAPeV is phylogenetically close to the porcine pestiviruses Linda virus and Bungowannah virus and causes similar pathological damage in pigs, its ORF nucleotide and amino acid identities with these viruses are less than 80%. These findings indicate that PAAPeV is an emerging pestivirus that poses a new threat to the swine industry and deserves increased attention. Clinically, monitoring and control of PAAPeV should be strengthened. More importantly, PAAPeV shares the highest sequence identity with Wenzhou *Pipistrellus abramus* pestivirus, suggesting a possible connection between them. We hypothesize that PAAPeV likely evolved from Wenzhou *Pipistrellus abramus* pestivirus and was transmitted from bats to pigs. Bats, known for frequent interactions with domestic animals, can act as vectors, transmitting pathogens among humans, domestic animals and wildlife, leading to the widespread dissemination of many infectious agents [26]. Several viral pathogens affecting humans and domestic animals, such as coronaviruses and hantaviruses, have been traced back to bats or transmitted by them, highlighting bats’ crucial role as reservoirs of viruses with significant public health implications [27–31]. Consequently, enhanced control and prevention measures targeting bats on pig farms are essential. In the future, further epidemiological investigations and retrospective studies will be necessary to accurately define the lineage of PAAPeV.

In vitro experiments revealed that PAAPeV exhibited limited replication in A549 and HepG2 cells, which ceased after 2 generations. However, in Vero cells, replication lasts for up to 6 generations. A previous report revealed similar behavior in PCV3, which, despite limited in vitro replication, retains the ability to replicate in pigs and is associated with various diseases [32]. Therefore, although PAAPeV demonstrated restricted replication in human and monkey cells, the possibility of infection in these species cannot be ruled out. Given the previously reported broad host range of other pestiviruses [33, 34], we believe that PAAPeV poses a potential threat to animal health and public safety.

Because PAAPeV does not cause CPEs in ST cells, its viral titre can only be measured via IFA, similar to CSFV [35]. Additional time is required to prepare the monoclonal antibody for PAAPeV, which has not yet been successfully produced. Therefore, the viral titre of PAAPeV remains undetermined. There are many future research areas worth exploring regarding PAAPeV, including its prevalence, persistent infection, mechanisms of pathogenesis and cross-species transmission capabilities.

In this study, we characterized a novel pestivirus in pigs, PAAPeV, and proposed its classification along with Wenzhou *Pipistrellus abramus* pestivirus as a new species within the *Pestivirus* genus *Pestivirus chinensis*. PAAPeV was isolated from aborted fetuses and piglets showing congenital tremors. It has demonstrated replication capabilities both in vitro and in vivo, causing pathological changes in piglets and mice. Additionally, PAAPeV exhibited varying degrees of susceptibility in the ST, A549, HepG2, and Vero cell lines. We hypothesize that PAAPeV likely evolved from Wenzhou *Pipistrellus abramus* pestivirus and has the potential for cross-species transmission, posing a threat to various animals and public health. As a newly discovered virus, further investigation is needed to determine its prevalence, mechanisms of pathogenesis, and cross-species transmission capabilities.

## Supplementary Information

**Additional file 1.** **Clinical symptoms and organ lesions in pigs naturally infected with PAAPeV.** **A** The sow experienced abortion and delivered stillborn piglets. **B** The sow delivered mummified piglets. **C** Piglets exhibited congenital tremors and difficulty standing. **D-F** Images of the lungs, spleen and liver of piglets naturally infected with PAAPeV.

**Additional file 2.** **Metagenomic sequencing analysis of viruses in lung and brain samples from piglets naturally infected with PAAPeV.** The results are shown at the family, genus, and species levels from left to right.

**Additional file 3.** **Establishment of a TaqMan-based RT-qPCR assay for specific detection of PAAPeV.** **A** Amplification curve for the PAAPeV NS3 gene-positive plasmid. Samples 1-5 represent a 10-fold serial dilution of the positive plasmid, ranging from 10^-2^ to 10^-6^. The x-axis shows cycle numbers, whereas the y-axis shows fluorescence intensity. **B** Standard curve of the PAAPeV NS3 gene-positive plasmid, with lg-transformed template concentrations on the x-axis and cycle threshold (Ct) values on the y-axis.

**Additional file 4.** **Preparation of mouse antiserum against the PAAPeV E2 protein.** **A** SDS‒PAGE analysis of PAAPeV E2 protein expressed in BL21 cells. M: marker; 1: pClod-TF plasmid; 2: supernatant of the recombinant plasmid pClod-TF-E2; 3: precipitate of the recombinant plasmid pClod-TF-E2; 4--7: E2 protein purified via Ni-NTA affinity chromatography. **B** Western blot analysis using mouse antiserum against the PAAPeV E2 protein as the primary antibody. M: marker; 1: E2 protein; 2: pClod-TF plasmid. The size of the E2 protein is indicated by the black arrow.

**Additional file 5.** **Reference pestivirus sequences used in the phylogenetic analysis for this study**.

**Additional file 6.** **ORF sequence identity analysis of PAAPeV with 51 representative pestiviruses.** **A** Nucleotide sequence identity analysis of the complete ORF was conducted using MegAlign software. **B** Amino acid sequence identity analysis of the complete ORF was performed using MegAlign software. The red boxes highlight the pestiviruses with the highest identities to PAAPeV, along with their corresponding identity values.

**Additional file 7.** **Multiple sequence alignment of the 5' UTR and 3ʹ UTR sequences of PAAPeV compared with those of Wenzhou *Pipistrellus abramus* pestivirus, Linda virus, Bungowannah virus and classical swine fever virus (CSFV).** **A** Multiple sequence alignment of the 5' UTR sequences was performed using the ClustalW method in MEGA software. **B** Nucleotide sequence identity of the 5' UTR sequences analysed using the ClustalW method in MegAlign software. **C** Multiple sequence alignment of the 3' UTR sequences was performed using the ClustalW method in MEGA software. **D** Nucleotide sequence identity of the 3' UTR sequences analysed using the ClustalW method in MegAlign software. The red boxes emphasize the identities between PAAPeV and other pestiviruses.

**Additional file 8.** **Organs of piglets infected with PAAPeV.** **A-G** represent the brain, lungs, liver, heart, spleen, kidney and lymph nodes, respectively.

**Additional file 9.** **Organs of mice infected with PAAPeV. A-F** represent the brain, heart, lungs, spleen, kidney and liver, respectively.

## Data Availability

The data supporting the findings of this study are available upon request from the corresponding authors.
