# Supplementary figures and images for: Emergence of a novel porcine pestivirus with potential for cross-species transmission in China, 2023
Source: Vet Res. 2025 Feb 7;56:32. doi: 10.1186/s13567-025-01472-5 (PMC11804013; doi:10.1186/s13567-025-01472-5)

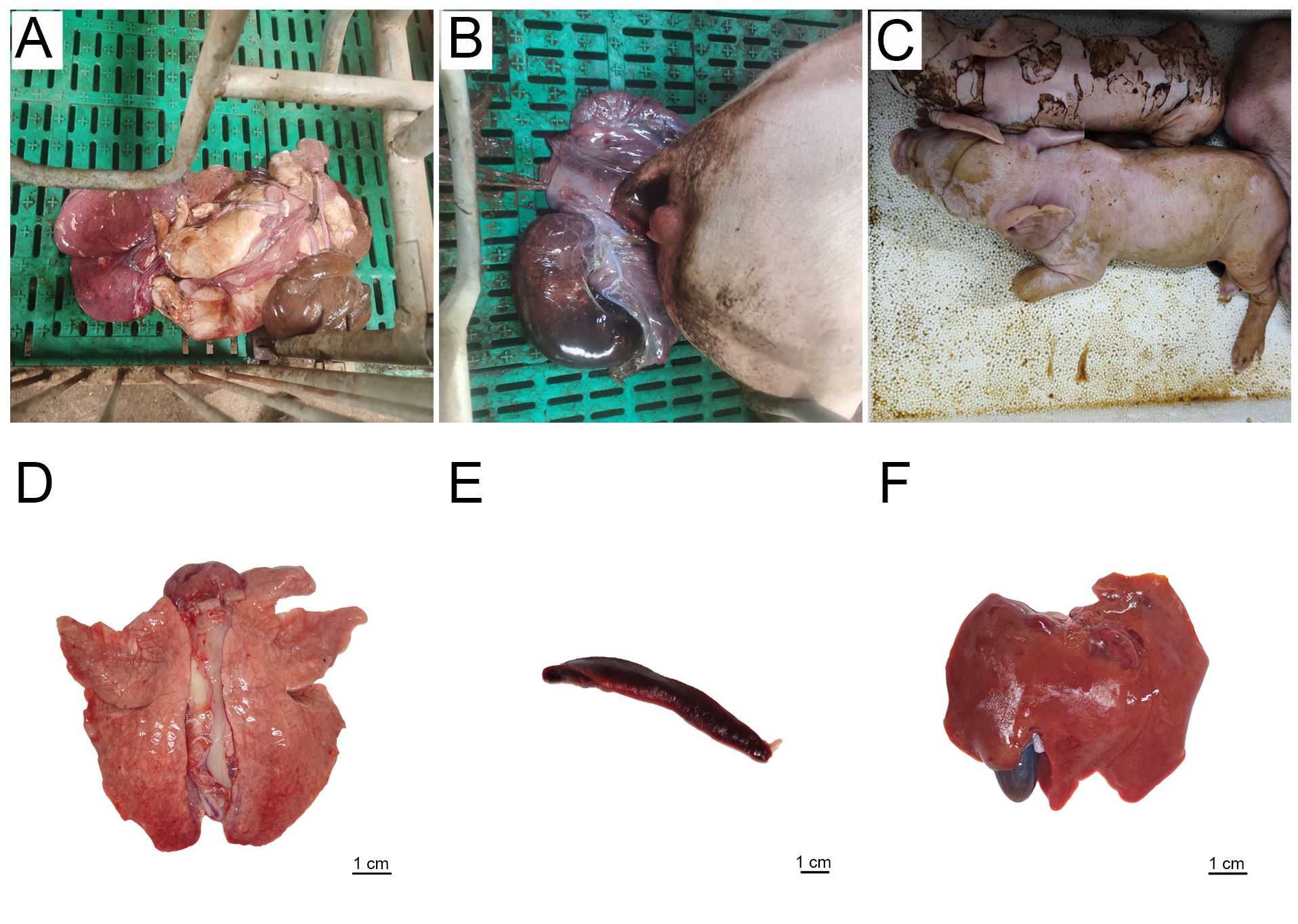

Supplement: Supplementary file 1 — Additional file 1. Clinical symptoms and organ lesions in pigs naturally infected with PAAPeV. A The sow experienced abortion and delivered stillborn piglets. B The sow delivered mummified piglets. C Piglets exhibited congenital tremors and difficulty standing. D-F Images of the lungs, spleen and liver of piglets naturally infected with PAAPeV. [file 13567_2025_1472_MOESM1_ESM.tif]

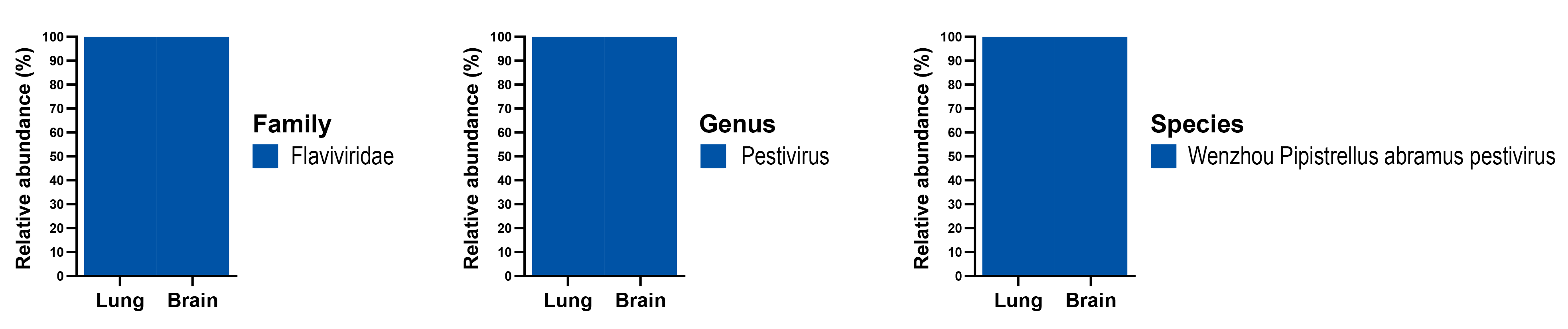

Supplement: Supplementary file 2 — Additional file 2. Metagenomic sequencing analysis of viruses in lung and brain samples from piglets naturally infected with PAAPeV. The results are shown at the family, genus, and species levels from left to right. [file 13567_2025_1472_MOESM2_ESM.tif]

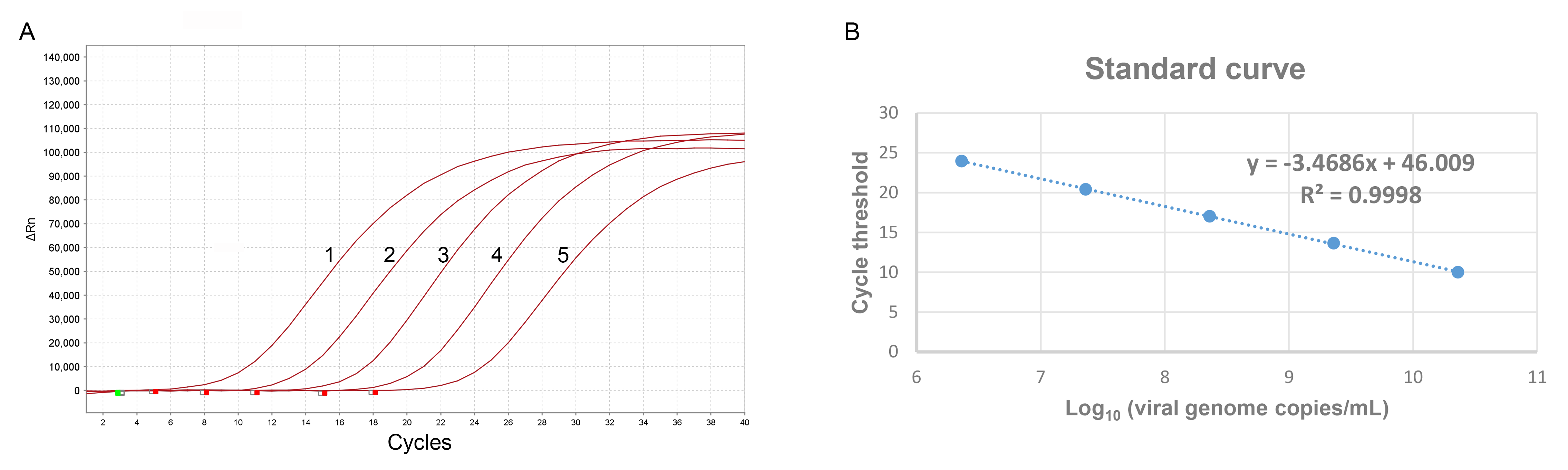

Supplement: Supplementary file 3 — Additional file 3. Establishment of a TaqMan-based RT-qPCR assay for specific detection of PAAPeV. A Amplification curve for the PAAPeV NS3 gene-positive plasmid. Samples 1-5 represent a 10-fold serial dilution of the positive plasmid, ranging from 10-2 to 10-6. The x-axis shows cycle numbers, whereas the y-axis shows fluorescence intensity. B Standard curve of the PAAPeV NS3 gene-positive plasmid, with lg-transformed template concentrations on the x-axis and cycle threshold (Ct) values on the y-axis. [file 13567_2025_1472_MOESM3_ESM.tif]

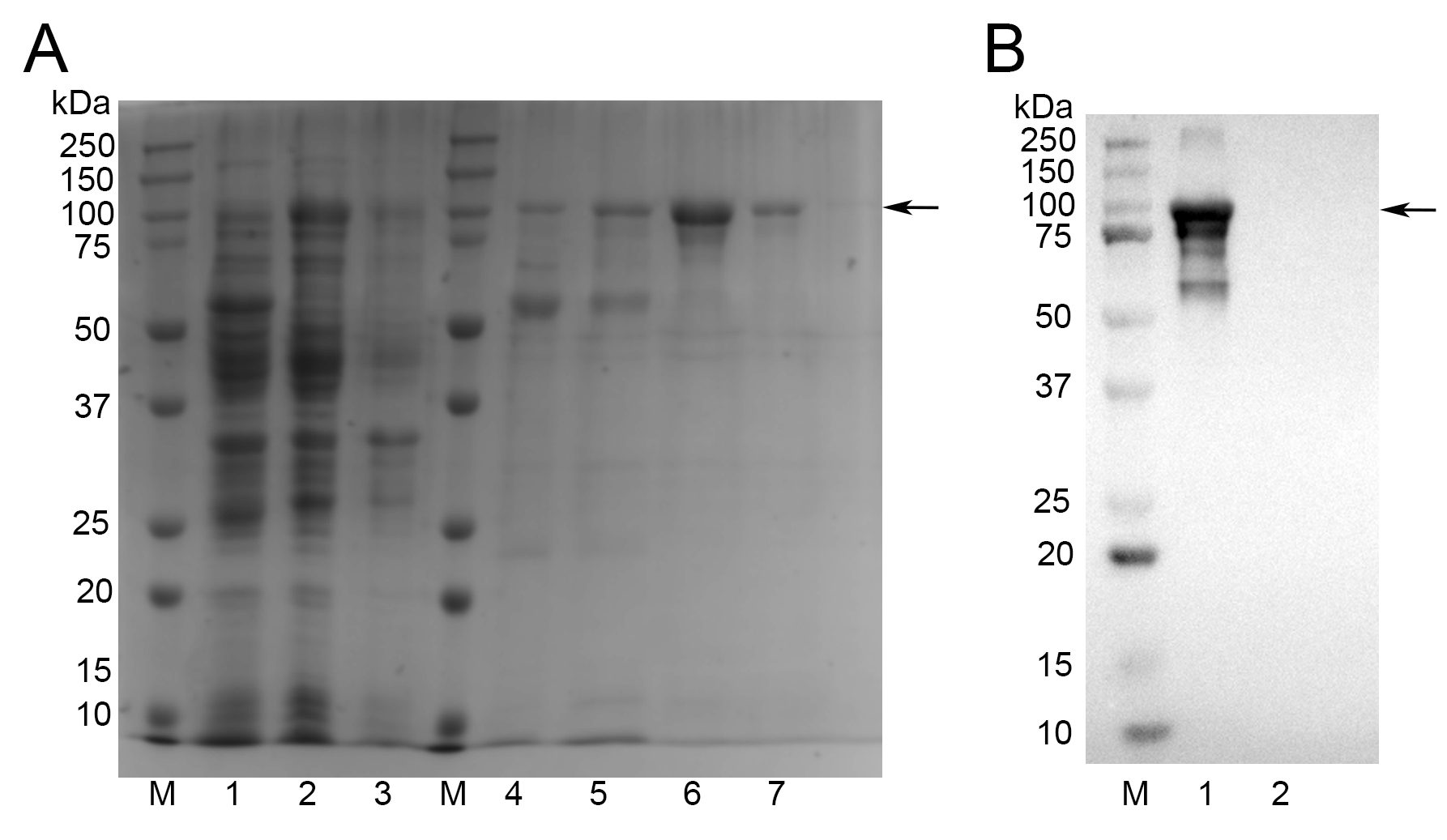

Supplement: Supplementary file 4 — Additional file 4. Preparation of mouse antiserum against the PAAPeV E2 protein. A SDS‒PAGE analysis of PAAPeV E2 protein expressed in BL21 cells. M: marker; 1: pClod-TF plasmid; 2: supernatant of the recombinant plasmid pClod-TF-E2; 3: precipitate of the recombinant plasmid pClod-TF-E2; 4--7: E2 protein purified via Ni-NTA affinity chromatography. B Western blot analysis using mouse antiserum against the PAAPeV E2 protein as the primary antibody. M: marker; 1: E2 protein; 2: pClod-TF plasmid. The size of the E2 protein is indicated by the black arrow. [file 13567_2025_1472_MOESM4_ESM.tif]

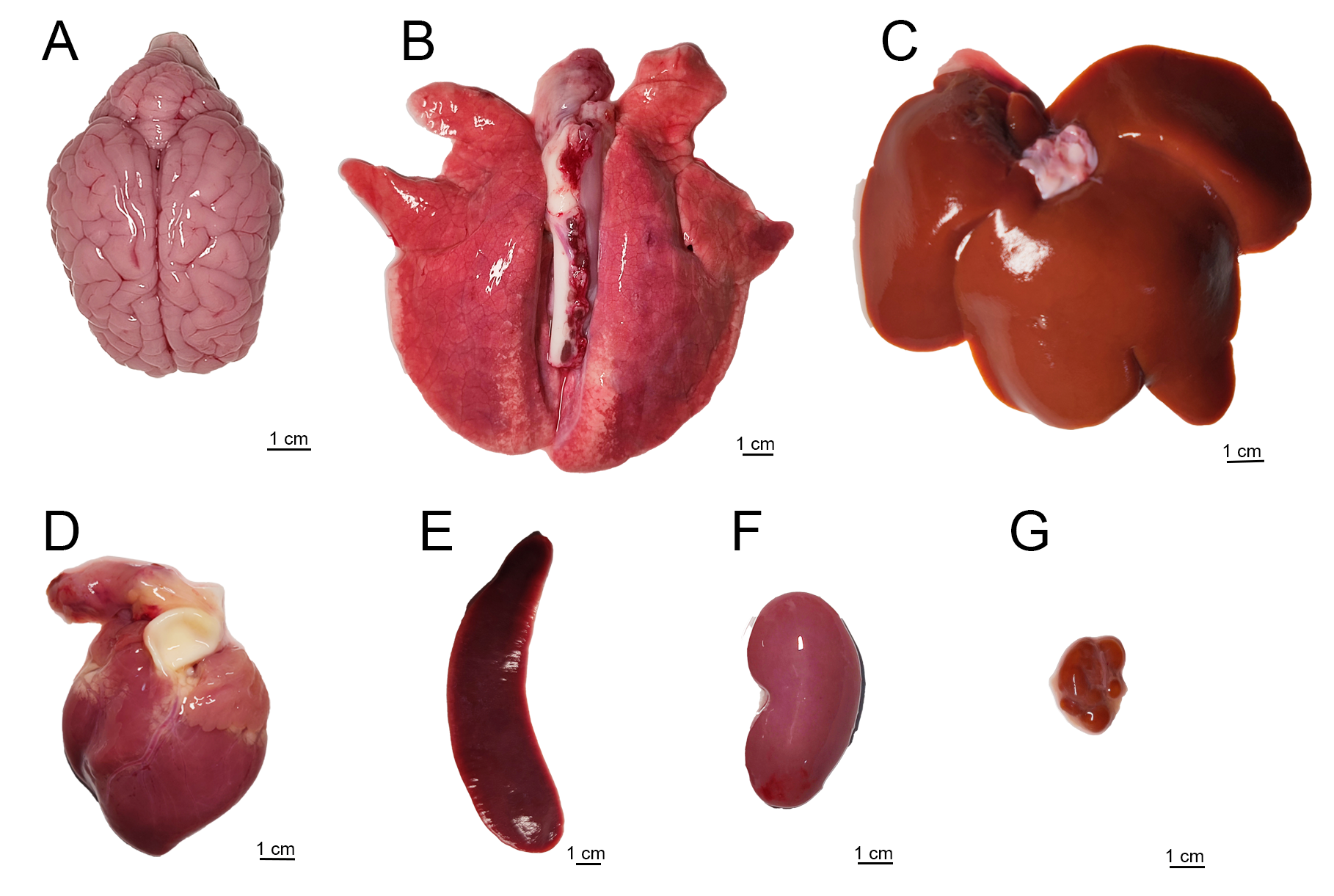

Supplement: Supplementary file 8 — Additional file 8. Organs of piglets infected with PAAPeV. A-G represent the brain, lungs, liver, heart, spleen, kidney and lymph nodes, respectively. [file 13567_2025_1472_MOESM8_ESM.tif]

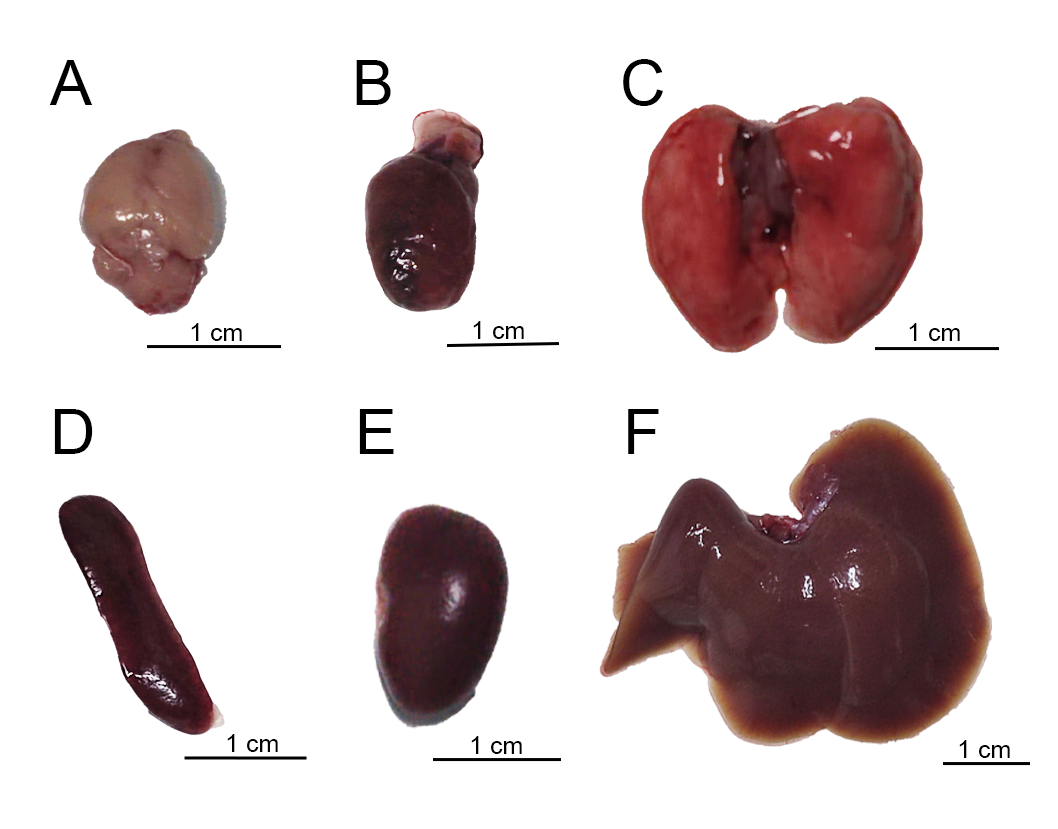

Supplement: Supplementary file 9 — Additional file 9. Organs of mice infected with PAAPeV. A-F represent the brain, heart, lungs, spleen, kidney and liver, respectively. [file 13567_2025_1472_MOESM9_ESM.tif]
