# Supplementary material for: Emergence of a novel porcine pestivirus with potential for cross-species transmission in China, 2023
Source: Vet Res. 2025 Feb 7;56:32. doi: 10.1186/s13567-025-01472-5 (PMC11804013; doi:10.1186/s13567-025-01472-5)
Supplement: Supplementary file 5 — Additional file 5. Reference pestivirus sequences used in the phylogenetic analysis for this study. [file 13567_2025_1472_MOESM5_ESM.docx]

**Additional file 5 Reference pestivirus sequences used in the phylogenetic analysis for this study.**

| **Species** | **Virus name** | **GenBank accession number** | **Host species** | **Length (bp)** |
| --- | --- | --- | --- | --- |
| *Pestivirus bovis* | Bovine viral diarrhea virus 1 strain SD0803 | JN400273.1 | Pig | 12 271 |
|  | Bovine viral diarrhea virus 1 strain ZM-95 | AF526381.3 | Pig | 12 220 |
|  | Bovine viral diarrhea virus 1 strain Shitara/02/06 | LC089876.1 | Bos taurus | 12 266 |
|  | Bovine viral diarrhea virus 1 strain SuwaCp | KC853441.1 | Bovine | 12 355 |
|  | Bovine viral diarrhea virus 1 strain Bega-like | KF896608.1 | Bovine | 12 193 |
| *Pestivirus tauri* | Bovine viral diarrhea virus 2 strain XJ-04 | FJ527854.1 | Cattle | 12 284 |
|  | Bovine viral diarrhea virus 2 strain KZ-91-CP | LC006970.1 | Bos taurus | 12 654 |
|  | Bovine viral diarrhea virus 2 strain HLJ-10 | JF714967.1 | Cattle | 12 284 |
|  | Bovine viral diarrhea virus 2 strain JZ05-1 | GQ888686.2 | Cattle | 12 285 |
|  | Bovine viral diarrhea virus 2 strain SD1301 | KJ000672.1 | Cattle | 12 258 |
| *Pestivirus suis* | Classical swine fever virus strain Bergen | KJ619377.1 | Pig | 12 295 |
|  | Classical swine fever virus strain Zj0801 | FJ529205.1 | Swine | 12 296 |
|  | Classical swine fever virus strain Alfort/Tuebingen | J04358.2 | cDNA clone | 12 297 |
|  | Classical swine fever virus strain 39 | AF407339.1 | Pig | 12 297 |
|  | Classical swine fever virus strain 94.4/IL/94/TWN | AY646427.1 | Pig | 12 296 |
|  | Hog cholera virus strain HCLV | AF091507.1 | Pig | 12 310 |
| *Pestivirus ovis* | Border disease virus strain Coos Bay 5 nc | KJ463422.1 | Ovis aries | 12 300 |
|  | Border disease virus strain FNK2012-1 | AB897785.1 | Sus scrofa domesticus | 12 327 |
|  | Border disease virus strain Gifhorn | KF925348.1 | Pig | 12 325 |
|  | Border disease virus strain H2121 (Chamois-1) | GU270877.1 | Rupicapra rupicapra (chamois) | 12 305 |
|  | Border disease virus strain Aveyron | KF918753.1 | Sheep | 12 284 |
| *Pestivirus antilocaprae* | Pronghorn antelope pestivirus | AY781152.3 | Pronghorn antelope | 12 273 |
| *Pestivirus australiaense* | Porcine pestivirus strain Bungowannah | EF100713.2 | Pig | 12 656 |
| *Pestivirus giraffae* | Pestivirus strain giraffe-1 H138 | AF144617.2 | Giraffa camelopardalis | 12 602 |
|  | Pestivirus strain PG-2 | KJ660072.1 | Bos taurus (cattle) | 12 264 |
| *Pestivirus brazilense* | Bovine viral diarrhea virus 3 strain D32/00_'HoBi' | AB871953.1 | Bos taurus | 12 265 |
|  | Bovine viral diarrhea virus 3 strain Italy-83/10-ncp | JQ612704.1 | Bos taurus | 12 243 |
|  | Bovine viral diarrhea virus 3 strain Italy-1/10-1 | HQ231763.1 | Cattle | 12 104 |
|  | Bovine viral diarrhea virus 3 strain Italy-129/07 | KC788748.1 | Bos taurus | 12 118 |
|  | Bovine viral diarrhea virus 3 strain LVRI/cont-1 | KC297709.1 | Bovine | 12 282 |
| *Pestivirus aydinense* | Pestivirus strain Burdur/05-TR | KM408491.1 | Goat | 12 281 |
|  | Pestivirus strain Aydin/04-TR | JX428945.1 | Sheep | 12 292 |
| *Pestivirus ratti* | Pestivirus ratti strain NrPV/NYC-D23 | KJ950914.1 | Rattus norvegicus | 12 983 |
| *Pestivirus scrofae* | Porcine pestivirus 1 strain ISDVDL2014016573 | KU194229.1 | Porcine | 11 545 |
|  | Porcine pestivirus 1 strain APPV_GER_01 | LT594521.1 | Porcine | 11 467 |
|  | Porcine pestivirus 1 strain 000515 | KR011347.1 | Porcine | 11 276 |
|  | Atypical porcine pestivirus strain NL1 Farm1 | KX929062.1 | Porcine | 11 561 |
| *Pestivirus L* | Linda virus strain Austria1 | NC_035432.1 | Swine | 12 614 |
|  | Linda virus strain Austria2 | KY436034.1 | Swine | 12 614 |
| *Pestivirus M* | Phocoena pestivirus strain NS170385 | MK910227.1 | Harbor porpoise | 11 880 |
|  | Phocoena pestivirus strain NS170386 | MK910228.1 | Harbor porpoise | 11 880 |
| *Pestivirus N* | Tunisian small ruminant pestivirus strain 70282/2007/EN | MZ664273.1 | Sheep | 12 234 |
|  | Tunisian small ruminant pestivirus strain 92019/2007/AG | MZ664274.1 | Goat | 12 286 |
| *Pestivirus O* | Ovine/IT pestivirus strain ovine/It/338710-3/2017 | MK618726.1 | Ovine | 12 173 |
|  | Pestivirus *sp.* strain Ovine/IT/1756/17 | MG770617.1 | Ovine | 12 173 |
| *Pestivirus P* | Dongyang pangolin virus strain DYAJ1 | MK636874.1 | Amblyomma javanense | 12 443 |
|  | Dongyang pangolin virus strain DYCS | MK636875.1 | Manis javanica | 12 446 |
| *Pestivirus Q* | Rodent pestivirus strain RtNn-PestV/HuB2014 | KY370101.1 | Niviventer niviventer | 13 220 |
| *Pestivirus R* | Rodent pestivirus strain RtAp-PestV/JL2014 | KY370100.1 | Apodemus peninsulae | 12 768 |
| *Pestivirus S* | Bat pestivirus strain BtSk-PestV-1/GX2017 | MH282908.1 | Scotophilus kuhlii | 11 921 |
| *Pestivirus chinensis* | Wenzhou *Pipistrellus abramus* pestivirus 1 strain YJB_Pabr | OM030320.1 | Pipistrellus abramus | 11 933 |
